# The HCV Synthesis Project: Scope, methodology, and preliminary results

**DOI:** 10.1186/1471-2288-8-62

**Published:** 2008-09-14

**Authors:** Rebecca K Stern, Holly Hagan, Corina Lelutiu-Weinberger, Don Des Jarlais, Roberta Scheinmann, Shiela Strauss, Enrique R Pouget, Peter Flom

**Affiliations:** 1 Center for Drug Use and HIV Research, National Development and Research Institutes (NDRI), 71 West 23rd Street, 8th Floor, New York, NY 10010, USA; 2 Baron Edmond de Rothschild Chemical Dependency Institute, Beth Israel Medical Center, 160 Water Street, 24th Floor, New York, NY 10038, USA; 3 Medical and Health Research Association, 220 Church Street, 5^th ^Floor, New York, NY 10013, USA; 4 Muriel and Virginia Pless Center for Nursing Research, NYU College of Nursing, 246 Greene Street, 616E, New York, NY 10003, USA

## Abstract

**Background:**

The hepatitis C virus (HCV) is hyper-endemic in injecting drug users. There is also excess HCV among non-injection drug users who smoke, snort, or sniff heroin, cocaine, crack, or methamphetamine.

**Methods:**

To summarize the research literature on HCV in drug users and identify gaps in knowledge, we conducted a synthesis of the relevant research carried out between 1989 and 2006. Using rigorous search methods, we identified and extracted data from published and unpublished reports of HCV among drug users. We designed a quality assurance system to ensure accuracy and consistency in all phases of the project. We also created a set of items to assess study design quality in each of the reports we included.

**Results:**

We identified 629 reports containing HCV prevalence rates, incidence rates and/or genotype distribution among injecting or non-injecting drug user populations published between January 1989 and December 2006. The majority of reports were from Western Europe (41%), North America (26%), Asia (11%) and Australia/New Zealand (10%). We also identified reports from Eastern Europe, South America, the Middle East, and the Caribbean. The number of publications reporting HCV rates in drug users increased dramatically between 1989 and 2006 to 27–52 reports per year after 1998.

**Conclusion:**

The data collection and quality assurance phases of the HCV Synthesis Project have been completed. Recommendations for future research on HCV in drug users have come out of our data collection phase. Future research reports can enhance their contributions to our understanding of HCV etiology by clearly defining their drug user participants with respect to type of drug and route of administration. Further, the use of standard reporting methods for risk factors would enable data to be combined across a larger set of studies; this is especially important for HCV seroconversion studies which suffer from small sample sizes and low power to examine risk factors.

## Background

Hepatitis C virus infection is hyper-endemic among injection drug user (IDU) populations [1,2], and evidence also suggests that excess HCV infection occurs in non-injection drug users (NIDUs) who administer heroin, cocaine, or amphetamine by other routes, such as inhalation or smoking [3,4]. HCV is a significant cause of morbidity and mortality in IDUs [5,6], and a leading cause of death among IDUs co-infected with HIV [7,8].

Once the hepatitis C virus was identified in 1989, the availability of serologic testing for HCV antibody (anti-HCV) led to a large number of studies of anti-HCV prevalence in IDUs during the 1990s. Four qualitative reviews of HCV epidemiology in IDUs were published by 2002; each summarized between 20 and 100 studies [1,2,9,10]. These reviews noted relatively high incidences, ranging between 6 and 40 seroconversions per 100 person-years (PY) of follow-up; the median incidence rates across the four reviews were 9, 12, 16, and 16/100 PY. Anti-HCV prevalence varied substantially, between 30 and 90%. Some of this variation was clearly related to time at risk, with a fairly consistent and relatively linear relationship between number of years injecting and increasing anti-HCV prevalence [11]. Geographic variability was also noted in these reviews, for example, higher mean HCV prevalence was found in studies of North American IDUs (82%), as compared to European (67%) or Australian/New Zealand IDUs (59%) [9]. Study year was also related to HCV prevalence, with the gradual emergence of reports of low-prevalence IDU samples in the literature. Altogether, research suggested that HCV infection varied in IDU populations in relation to characteristics of person, place and time, and that insights into HCV prevention might be obtained via a systematic, quantitative review of available studies.

Fewer studies of HCV epidemiology have been conducted among NIDUs than among IDUs, and the etiology of HCV transmission in this population is not as well-understood, although some believe that exposure to HCV-positive blood occurs as a result of the shared use of pipes or straws to administer the drug [12]. Excess HCV in NIDUs has been shown in a number of studies, with HCV prevalence reported between 1 and 35% [13], compared to 1.8% anti-HCV prevalence in the general population [14]. Very few NIDU studies have closely examined route of HCV exposure, principally because they used a cross-sectional design, thus weakening the interpretation of a causal link between risk factors and incident infection. Consistency in findings across studies, or consistency in explanation for variation across studies, may support an underlying causal relation between these forms of drug use and HCV.

A recent qualitative review showed that studies of strategies to prevent HCV infection in IDUs had inconsistent results, with few examples of interventions that reduce HCV transmission. Moreover, the majority of studies had small sample sizes [11]. Most HCV prevention efforts thus far have used strategies shown to decrease HIV transmission in drug users, including drug treatment, voluntary counseling and testing, and needle exchange programs. It appears that their effect on HCV infection may be attenuated by several factors, particularly that HCV is more highly prevalent than HIV in IDU populations so that the probability of injecting with an HCV-infectious IDU is greater than injection with an HIV-positive injector. Additionally, there are many more materials used to inject or prepare drugs that may transmit HCV infection in the injection setting. Specifically, the shared use of syringes has been demonstrated to transmit HIV and HCV, but other equipment used to prepare drugs for injection (drug cookers and filtration cotton) may also transmit HCV [15-18]. Nonetheless, despite the lack of evidence showing an individual-level effect of various prevention strategies on risk of HCV infection, declining HCV prevalence has been observed in settings where comprehensive HIV prevention for drug users is widely available (such as large-scale syringe exchange and access to drug treatment) [19,20]. Altogether, the published literature suggested that a synthesis of research on the epidemiology and prevention of HCV in drug user populations was needed to examine etiologic factors and drug or sexual practices that may reduce risk of HCV transmission, and to identify gaps in the literature.

This article describes the scientific scope of this systematic research synthesis study, the criteria used to select reports to include in the study, the methodology used to identify and code relevant reports, the system employed to assure accuracy and consistency in all phases of the project, and a summary of the study sample. Other phases of the research synthesis study are also described. Finally, our protocol for evaluating indicators of study quality and the studies in our sample are summarized.

### Scope of the project

We undertook the task of systematically reviewing all studies describing the epidemiology of hepatitis C in drug-user populations to address questions regarding factors that are associated with variability in HCV transmission. The scope of this study, "Synthesis: A meta-analysis of HCV epidemiology and prevention in drug users" (the HCV Synthesis Project) encompasses published and unpublished reports from the US and abroad describing the epidemiology of HCV infection (incidence and prevalence), the molecular epidemiology of HCV genotypes, and the co-occurrence of HIV, HCV and other hepatitis virus infections in drug users. Measures of association between HCV prevalence and incidence and factors such as risk behavior and participation in prevention programming are also collected. The population of interest includes injection drug users, and non-injection drug users of heroin, cocaine and amphetamine, because these groups of individuals have been identified as having a biologically plausible risk of exposure to HCV via drug use, including percutaneous exposure (via injection drug use) and mucous membrane exposure to HCV-positive blood via sharing of straws or pipes used to administer drugs (non-injection drug use). Data from IDU and NIDU studies are analyzed separately, as they differ substantially in the likelihood of HCV infection, and because their drug-related risk factors are quite dissimilar. In addition, a higher proportion of HCV infections in NIDUs vs. IDUs may be attributable to sexual rather than drug-related exposures, because there is less evidence to support specific drug-related transmission among NIDUs [22].

Another goal of the meta-analysis is to examine the influence of study methodology on study findings, particularly because the descriptive epidemiology of HCV may be strongly influenced by sampling methods, and because study design may affect associations between various characteristics and HCV infection. Since HCV antibody testing is a recent development (1989), it is possible to carry out a relatively complete synthesis covering approximately 18 years (1989–2006) of research studies that have reported incidence, prevalence and measures of association with HCV infection in drug users. Consistent with the fundamental goals of meta-analysis, the purpose of this study is to generate summary data, describe variation in HCV epidemiology, resolve inconsistencies in findings, and identify areas of future research.

## Methods

### Search strategy

Automated searches of published literature were carried out on electronic databases (MEDLINE, PsychInfo, ERIC, Dissertation Abstracts, Sociological Abstracts, and Current Contents) using the following search terms: (hepatitis C OR HCV) AND (intravenous drug abuse OR intravenous drug use OR drug misuse OR drug addict OR injecting drug use OR drug abuse OR IDU) AND (prevention OR risk factor OR epidemiology OR prevalence OR incidence OR seroprevalence OR seroincidence OR genotype OR co-infect* OR coinfect*). We performed searches in these electronic databases at six month intervals.

Manual search methods included the retrieval of sources cited by seminal articles about HCV in drug-user populations, and hand searching of journals. We compiled a list of scientific journals that have either published articles on HCV epidemiology and prevention, or might conceivably publish such articles based on a history of publishing articles in a similar field (HIV epidemiology, for example). Internet searches of government websites (including local, state and national public health websites in the US; provincial, national and ministry of health websites in other countries; and websites for international health or drug control organizations) were carried out to locate government reports and unpublished surveillance estimates.

Books of abstracts and proceedings from scientific conferences related to hepatitis, HIV, infectious diseases and harm reduction were also searched for eligible reports, using both the index of key words and by reading through the abstracts of presentations. The NIH CRISP database was also used to identify ongoing or recently completed studies relevant to our meta-analysis; names of investigators identified in CRISP were periodically entered into the electronic databases to identify publications that might conceivably report on measures of interest. Consultants to the study were enlisted to submit reports from their own studies or from other studies they had learned about through professional contacts at conferences or other meetings; these consultants included investigators who were carrying out research related to HCV in drug users in the US, Europe, Asia, Africa, the Middle East and Australia/New Zealand.

### Study selection

Throughout this description of study methods, we use the term data report to refer to published and unpublished articles, manuscripts, personal communication, dissertations, abstracts, conference presentations and book chapters that are reviewed for inclusion in the study sample. Data reports available through the end of 2006 were included in the search; study retrieval and coding began in August, 2004.

To be included in our study, data reports must have included in their sample individuals who could conceivably have acquired HCV infection via administration of an illegal drug, i.e., injection drug users, or non-injection drug users who snort, sniff or smoke heroin, cocaine, amphetamines or other drugs using straws or pipes. Marijuana smokers were not included in the study as there are no epidemiologic data to indicate excess HCV in marijuana smokers. Similarly, those who administered amphetamines or other illicit drugs orally (i.e., took pills) were also excluded as there is no biologically plausible route of HCV transmission associated with this practice.

Only those reports from which we could abstract estimates of HCV prevalence, incidence, measures of association, HIV/HCV co-infection or HCV genotype distributions for drug users were included. Further, reports must have given separate estimates for IDUs and NIDUs, as these rates were expected to vary greatly between these two risk groups. Thus, studies that aggregated IDUs and NIDUs in estimates of prevalence, incidence or measures of association were not included in this review. HCV status must have been determined by serologic testing of either sera or saliva; studies that used self-reported HCV antibody status, or those that tested for HCV RNA without reporting the results of anti-HCV testing were excluded.

Each data report could have one or more of the following types of studies associated with it: HCV prevalence studies, HCV incidence studies, HCV genotype studies or studies of HCV co-infection with HIV or other hepatitis viruses. Within a single report, each of these types of studies could appear and be counted as an individual study by our definition. A report was also defined as having multiple studies when epidemiologic estimates were given for subgroups of individuals distinguished by different methodology (e.g., enrollment criteria, sampling location or sampling method) or were reported as separate samples with different demographic characteristics or inclusion criteria. For example, a study in IDUs that spanned five years, and presented all their data (sample demographics and HCV prevalence) for each year of data collection was counted as five prevalence studies. Separating the data into individual studies permitted the collection of sample characteristics or methodological features associated with the subset of subjects being examined.

### Screening

A pilot study was carried out to test and develop procedures for screening titles and abstracts to identify data reports that would be eligible to be retrieved, and to estimate an expected sample size (the number of data reports that would provide data to address the aims). In October 2003, we conducted a Medline search using the keywords mentioned above; this search retrieved 1324 articles. Abstracts we obtained from a 5% random sample of these articles (n = 63) and three study staff (the PI, a co-investigator and a research assistant) independently pre-screened each title and abstract. Of necessity, the criteria for screening were broad so as to reduce the likelihood of missing relevant studies, but it was also desirable that screening have relatively high specificity to avoid retrieving a large number of irrelevant studies.

The screening criteria were: 1) sample included IDU or NIDU, and 2) study reported any parameters of epidemiology (incidence or prevalence of HCV-antibody or HCV-genotype), HIV/HCV co-infection, or measures of association. For this pilot, studies must also have been written in English. Initially, there was 80% agreement as to whether the article should be retrieved. Screening criteria were discussed and revised to reach consensus in their application.

Beginning in August 2004, we followed the process developed in the pilot study to screen abstracts to determine whether a report was eligible in terms of its sample and the data it provided. For the reports whose abstracts deemed them potentially eligible, the full text was reviewed to determine whether it truly met our inclusion criteria. Each abstract or report was screened for eligibility by both a senior research assistant and the project director. Screening sought to eliminate studies that were clearly unrelated to the scope of the project. In cases where it could not be definitively determined whether a report was eligible based on reading the abstract, the full text article was retrieved and reviewed for eligibility. When the full text of an article was not available in English, Spanish, or Italian, the English-language Medline abstract of the article was used for coding.

**Figure 1:**
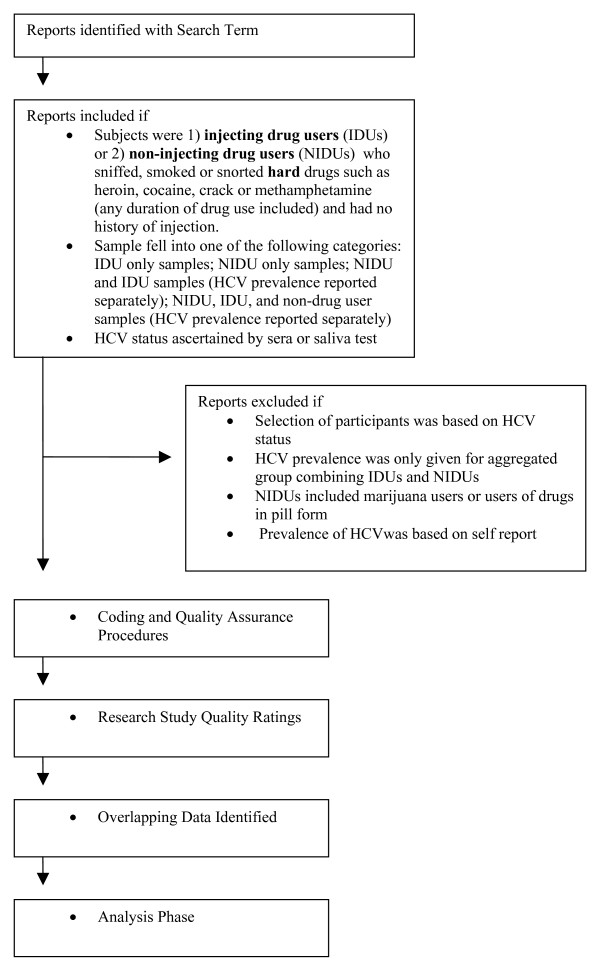
HCV Meta-Analysis Project Phases.

It was common to find multiple HCV-related reports originating from a single large research project such as ALIVE, VIDUS, CIDUS, RAVEN and many others [23-30]. Thus, several methods were used to identify duplicate or overlapping reports (for example, recording study names and searching our reference manager database for other reports by the same author or set of authors). These potentially-overlapping reports were identified but retained in the database, as some reports included analyses of different sets of factors or subsets of the study sample. Duplicate estimates of the same parameters for the same datasets will be excluded prior to each data analysis project. (Figure 1 shows a decision tree of our study selection and screening process.)

### Coding

The coding was carried out by senior research assistants who had graduate training in research methodology, and received additional training in HCV epidemiology, drug use and meta-analytic methods. The content and structure of the coding form was developed by reviewing those used in other meta-analyses, for example, the CDC Prevention Research Synthesis Project [31] and the Self-Report/Biological Measures database of Drug Use [32] The coding form included items such as the type of study or studies included in the body of the report (prevalence, incidence, co-infection or genotype study), study methods (design, inclusion criteria, recruitment method, recruitment locations, method of determining IDU/NIDU status, specimen type, and HCV test method), and demographics and other characteristics of the sample such as duration of drug use, type of drugs used, and frequency of use.

We collected all data related to HCV prevalence and incidence, i.e., all numerators and denominators, including both numbers and proportions of subjects and numbers of person-years, to allow us to combine data from separate studies in subsequent analyses. Anti-HCV prevalence and incidence estimates, relative risks and odds ratios (both crude and adjusted, with their 95% confidence intervals) were also collected in relation to sample characteristics. Simple calculations were made when necessary, and any approximations which were recorded (such as numbers estimated from reading a figure) were marked as such.

In some cases, the statistics recorded were simple measures of overall HCV prevalence or incidence for the entire eligible sample, while in other cases prevalence or incidence measures were reported within highly restricted subgroups defined by a number of variables such as age, gender, race and type of drug used. The coding form was structured so that we could abstract both simple and complicated overall estimates and sub-group comparisons, and therefore accommodate varying degrees of complexity in the data.

Thus, the coding form included fields for a number of pre-defined categories of gender, race/ethnicity, drug use, and risk behavior categories such as receptive and distributive syringe sharing, the shared use of drug preparation equipment (e.g., drug cooker, filtration cotton and rinse water), the use of a syringe to divide drugs, giving or receiving injections, injection in prison or jail, and participation in prevention activities such as drug treatment or syringe exchange programs. Sexual risk behavior data was also collected, for example, number of sexual partners, unprotected sex, commercial sex work and having an IDU sex partner. For each of these risk categories describing behavior, we recorded the referent time period used. There were also fields included in the coding form intended to capture relevant data that did not conform to our preset categories.

We did not record measures such as odds ratios comparing HCV prevalence or incidence in IDUs to that among NIDUs or non-drug users, as there is clear evidence that injection drug use is a highly potent risk behavior for HCV acquisition, and comparisons among such populations would not contribute new knowledge to our understanding of HCV epidemiology.

### Quality assurance methods

A number of strategies were used in the course of this study to ensure reliable, valid and consistent coding of data. For example, each data report coded by a research assistant was subsequently reviewed for accuracy and completeness by the Project Director and the Principal Investigator. The coders made any necessary changes to the coding before the report was considered complete. Any differences of opinion among the study group members were settled by discussion at a weekly study meeting convened for this purpose. A study manual was developed to guide coding and to record special cases and their resolution.

### Backcoding

Once a significant portion of the published data had been coded (October 2005), the research assistants conducted a data-coding reliability sub-study. A 10% sample of eligible coded reports was selected and re-coded by a different coder and all discrepancies in coding were noted and summarized. This project was undertaken to establish whether (1) any changes in coding rules had occurred over the course of the study, (2) there were any systematic differences between coders or (3) there were any specific items that may have been inconsistently coded. This reliability project revealed that two items (recruitment method and recruitment location) were inconsistently coded. Our review of the text of the reports revealed that variation between coders was principally due to a lack of clarity in the presentation of this information across a significant number of reports. Coding rules for these two variables were discussed and re-defined, and these items were back-coded for all articles.

### Contacting Authors

When the text or abstract of an article or conference presentation indicated that measures of interest to our study had been collected but were not reported, we contacted authors to request the information. These requests were limited to simple descriptive data such as separate estimates of HCV prevalence or incidence for IDUs vs. NIDUs, or to ask for the numerator or denominator used to estimate incidence or prevalence. We did not ask for additional analyses of data. Authors were contacted only if the report had been published or presented less than five years before the coding date. Overall, we contacted just over 100 authors, and 42% of authors we contacted sent a reply to the request for data. Of those who replied, 70% provided data that could be included as part of the meta-analysis.

### Unpublished data

We also searched subject indexes of conference proceedings (abstracts) on paper, CD-ROM or various websites. The conferences included were annual meetings of the American Association for the Study of Liver Diseases, the Society for Epidemiologic Research, the American Public Health Association, the International AIDS Society, the National Harm Reduction Conference, the International Conference on the Reduction of Drug Related Harm, the College on Problems of Drug Dependence, International Society for Infectious Diseases, and the American Sociological Association. Investigators from the study also attended a number of these conferences and collected copies of presentations and other unpublished reports. These abstracts were screened and the data were coded by the same process we used for published abstracts and articles.

Substantial effort was devoted to ascertaining whether the data in the unpublished literature duplicated any reports that had been previously coded. This step involved cross-checking of authors of unpublished studies against our database and in PubMed. When matches were found, the unpublished literature was compared to the published report to identify whether they overlapped in terms of location, number of subjects, and year of data collection. Approximately half of the conference abstracts that appeared to be codeable were subsequently eliminated because there was a published article with overlapping data.

### Study quality measures

We followed the recommendation of the MOOSE group [21] that meta-analyses of observational studies evaluate certain elements of each included study indicative of their quality. Thus, we devised a number of 'quality' items to assess whether each study's design and implementation was clearly explained and appropriate for the claims made in the reports. Our scale rated several study factors likely to affect findings of HCV prevalence, incidence and co-infection in drug user populations, including the rigor with which the investigators handled the problem of misclassification with respect to route of drug administration. Additionally, the quality scale assessed some aspects of the overall design and methodology of the studies, such as sample size, reporting of demographics, presence of inclusion or exclusion criteria, participation rate, adjustment for confounding, and consistency in reporting data. Sources of bias in study methods have been demonstrated to account for heterogeneity in results in meta-analyses [21].

An initial list of quality criteria was developed by the research team to assess NIDU studies included in a paper published by our group [13]. The quality criteria for the IDU sample were modeled on the NIDU quality scale. A detailed description of the NIDU quality scale can be found in Scheinmann et al. [13]. For the complete list of quality items used for evaluating IDU studies, see Table 1.

**Table 1:** Quality Measure

**Quality/rigor/relevance of IDU data in studies included in HCV meta-analysis**
**Type of study**

**Issues related to studying IDUs**
Was one of the stated aims to study the disease in IDUs or drug users (literature indicates interest to study HCV in IDUs)?
Sample composition (1 = non-drug users and drug users; 2 = NIDUs and IDUs; 3 = mostly IDUs; 4 = only IDUs)
Was there a method for minimizing misclassification bias (e.g., track marks or multiple interview questions)?

**Methodological issues**
Were dates of data collection given?
Were the selection criteria for the sample well defined and explained?

Were details of recruitment methods given?
Were details of recruitment location given?
Were there any incentives offered to the participants?
Were participation rates given for the IDU sample?
What was the participation rate?
Did data collection methods change during the study (e.g., recruitment method; face-to-face interviewing vs. self-administered questionnaire; testing method; etc)
Were the statistical methods used stated (for contrasts and/or measures of association)?
What was the IDU sample size tested for HCV prevalence (denominator)?
Were the number of subjects and percentages consistent?
Were age characteristics given for the IDU sample?
Were gender characteristics given for the IDU sample?
Were race/ethnic characteristics given for the IDU sample?
Were duration of injection data given for the IDU sample?

**Issues related to IDU-specific behaviors/characteristics**
Were there univariate analyses of prevalence?
Were there multivariate analyses of prevalence?
Was HIV prevalence given?

To determine whether the items in our quality scale were internally consistent we performed Cronbach's alpha reliability analyses using two-thirds of the data from the HCV Synthesis Study. The 19-item scale had an alpha value of 0.73, reflecting adequate scale coherence. Removal of items with low item-total correlations did not improve the alpha. The distribution of the quality scale scores was bimodal. Quality was then recoded dichotomously by splitting between the modes.

## Results

Overall, the HCV Synthesis Project has identified 2384 reports through the electronic search terms. Of those, 948 (40%) reports appeared to be eligible for coding. In most cases of ineligible reports, it was immediately apparent that the reports were review articles, were not reporting any original data, or did not report any data on either IDUs or NIDUs. 319 reports were initially classified as codeable, but were disqualified upon closer review. The main reasons for disqualification were 1) no HCV prevalence or incidence data (n = 71); 2) drug user prevalence data aggregated, i.e., IDU and NIDU estimates were not disaggregated or distinguished from non-drug users (n = 64); 3) sample selection based on HCV status (n = 56); 4) results based on HCV-RNA testing alone (n = 9); and 5) self-reported HCV status was basis for prevalence estimates and no lab testing was performed (n = 26). Other reasons for disqualification of reports were that no drug users were included in the report; data were incomplete for coding; the article included only a mathematical model; or the article was a review and did not include original data.

Thus, 629 codeable reports containing HCV prevalence rates, incidence rates and/or genotype distribution in injecting or non-injecting drug user populations published between January 1989 and December 2006 were identified. Each report contained one of more of the following types of information: prevalence rates, incidence rates, genotype distributions and/or coinfection information. Of the 629 eligible reports (some of which included more than one of the following types of statistics), 520 reported HCV prevalence statistics, 62 reported incidence statistics, and 118 reported genotype distribution. Further, 345 also reported co-infection with HIV, HBV or HAV.

Of note, a given report (e.g., a published article) may contain more than one study if it provides separate data for samples collected in different locations, time periods and/or with different demographic data. Therefore, one report may contain multiple studies, of either the same type (two prevalence studies, for example) or different types (a prevalence study and an incidence study, for example). We have 794 studies overall, consisting of 599 prevalence studies, 72 incidence studies and 123 genotype studies.

Fewer than 10% of the reports included data on individuals meeting our definition of NIDUs. More than 95% of all eligible reports were identified through Medline searches. (Note that these numbers of reports do not eliminate overlapping reports from single studies.)

**Figure 2:**
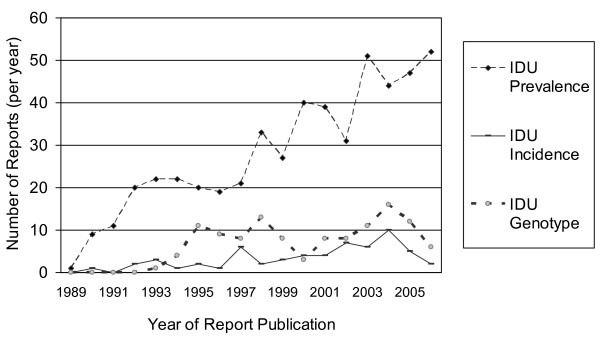
Number of reports per year describing HCV prevalence, incidence or genotype distribution in injection drug users in the HCV Synthesis Project.

As shown in figure 2, the number of publications reporting HCV prevalence in IDUs rose from 1–11 reports per year from 1989 to 1991, to 19–22 reports per year from 1992–1997, 33 reports in 1998, and between 27–52 reports per year after 1998.

The number of seroincidence reports in IDUs has risen more slowly and typically remained below seven per year, although in 2004 there were ten seroincidence reports. The number of HCV genotype studies among IDUs has ranged between 4 and 16 per year since 1994. The number of reports of HCV prevalence in NIDU samples per year has fluctuated between zero and ten. Genotype and seroincidence in NIDUs are rarely reported, with no more than one report in any given year (data not shown).

The majority of reports collected data from western Europe (41%), North America (26%), Asia (11%) and Australia/New Zealand (10%). We also identified reports which collected data from Eastern Europe (5%), South America (4%), the Middle East (2%) and the Caribbean (0.3%). For a listing of the distribution of reports in the synthesis sample by country, see Table 2.

**Table 2:** Number of Reports per Country

Geographical Location of Studies in HCV Synthesis Project		
Country	Number of Reports	Percentage
Argentina	4	0.6%
Australia	56	8.9%
Austria	8	1.3%
Bangladesh	4	0.6%
Belgium	7	1.1%
Bosnia and Herzegovina	1	0.2%
Brazil	20	3.2%
Bulgaria	2	0.3%
Canada	22	3.5%
China	17	2.7%
Croatia	2	0.3%
Czech Republic	5	0.8%
Denmark	3	0.5%
Egypt	1	0.2%
England	20	3.2%
Estonia	1	0.2%
Finland	2	0.3%
France	22	3.5%
Georgia	3	0.5%
Germany	19	3.0%
Greece	9	1.4%
Haiti	1	0.2%
Hong Kong	2	0.3%
Hungary	1	0.2%
Iceland	3	0.5%
India	7	1.1%
Iran	3	0.5%
Ireland	13	2.1%
Israel	2	0.3%
*Italy	47	7.5%
Japan	4	0.6%
Lebanon	2	0.3%
Malaysia	4	0.6%
Martinique	1	0.2%
Mexico	1	0.2%
Multiple	3	0.5%
Nepal	4	0.6%
New Zealand	8	1.3%
Norway	4	0.6%
Pakistan	1	0.2%
Philippines	1	0.2%
Poland	3	0.5%
Portugal	1	0.2%
Russia	11	1.8%
Saudi Arabia	4	0.6%
Scotland	20	3.2%
Slovenia	1	0.2%
*Spain	46	7.3%
Sweden	12	1.9%
Switzerland	7	1.1%
Syria	1	0.2%
Taiwan	9	1.4%
Thailand	13	2.1%
The Netherlands	10	1.6%
UK	3	0.5%
USA	139	22.2%
Uzbekistan	3	0.5%
Vietnam	3	0.5%
Wales	1	0.2%

* Since we coded articles in Spanish and Italian, as well as English language articles, reports from Italy and Spain may be over-represented in comparison to reports from other countries outside the US.

## Discussion

Electronic searches of the published literature retrieved a substantial number of reports on the epidemiology of HCV infection in drug users. The fact that nearly all eligible reports were discovered via Medline searches suggests that study data are relatively accessible for a meta-analysis. Our search of the unpublished data has so far retrieved relatively few additional reports. This is much higher than the 50% expected hit-rate using electronic databases [21]. Thus, exclusion of pertinent data due to publication bias or other types of systematic omissions from our study sample are unlikely to be of sufficient magnitude to substantially bias our findings. Funnel plots and other methods will be used to systematically examine publication bias (to judge whether studies with small samples and low HCV prevalence are missing from the literature).

The number of studies and reports related to HCV in injection drug users has been increasing over time, particularly since 1995. Cross-sectional prevalence studies make up the majority of reports we identified; these will be useful in terms of identifying characteristics of person, place and time associated with extreme rates of HCV prevalence. Because cross-sectional surveys are commonly used to characterize the epidemiology of HCV and other conditions in local drug user populations, understanding whether sampling methodology is associated with prevalence will help guide inferences regarding these data. The number of seroincidence reports has grown over recent years, and this relatively large sample (n = 62) should permit greater exploration of sources of variability in seroconversion rates. Synthesis of co-infection studies reporting HCV, HBV and HIV infection rates will provide insight into the joint occurrence of these infections and factors associated with similarities in their epidemiology in drug users. Co-infection data and HCV genotype information will be relevant to health care planning, particularly the treatment of HIV/HCV co-infection and HCV mono-infection in drug users.

**Figure 3:**
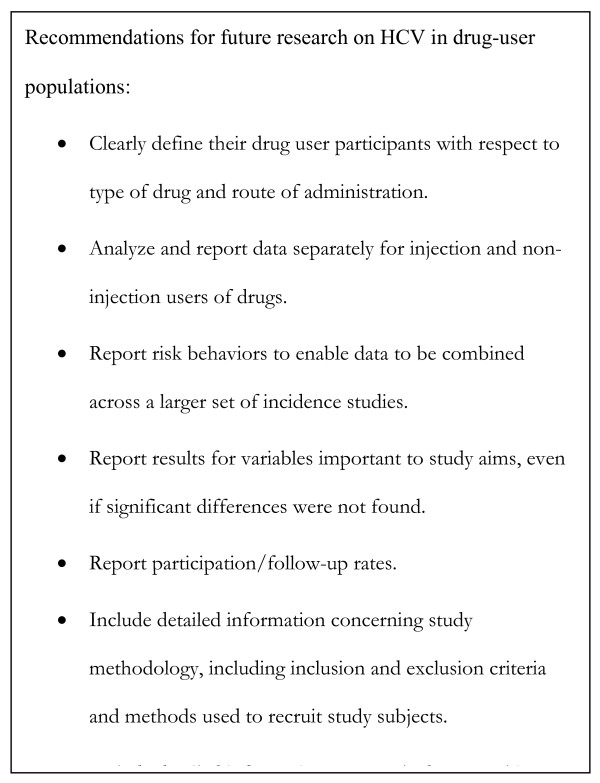
Recommendations for future research on HCV in drug-user populations.

The scope and aims of the HCV Synthesis Project differ from typical meta-analyses which seek to combine effect sizes arising from studies of interventions or treatments. Nonetheless, we adapted standard approaches to meta-analysis [33] to synthesize parameters of epidemiology. The challenges we encountered in collecting and coding these data include lack of standard reporting of risk factor data, overlapping publication of data, and missing details on study methodology. One notable finding from our summary of the HCV Synthesis Project data is that future research reports can enhance their contributions to our understanding of HCV epidmiology by clearly defining their drug user participants with respect to type of drug and route of administration, and by analyzing and reporting data separately in injection and non-injection users of drugs. (Figure 3 summarizes recommendations for future research on HCV among drug user populations.)

Had more studies disaggregated IDU and NIDU data, our sample of NIDU studies would have potentially doubled (provided that the NIDU sample fit our definition of non injection drug users who sniff, snort, or smoke hard drugs). Unfortunately, a large number of studies that were ineligible for this reason were from developing countries for which HCV data are scarce. The use of standard reporting methods, as promulgated by MOOSE [21], would enable data to be combined across a larger set of studies; this is especially important for HCV seroconversion studies which suffer from small sample sizes and low power to examine risk factors. MOOSE developed recommendations for meta-analyses of observational studies of interventions and etiologic factors. In general, we have followed their recommendations adapted to our specific research questions and the field of drug use related research. For example, we did use broad inclusion criteria which has yielded a large sample size.

Despite the fact that meta-analysis is traditionally used to combine results from randomized studies, such as randomized control trials, the use of meta-analysis to combine results from observational studies is becoming widespread because of the many issues in public health which cannot be studied without the use of observational designs. In order to apply meta-analytic techniques to sets of observational studies, it is necessary to develop homogeneous subsets, so that confounding factors do not overly influence the meta-analytic results [35]. Heterogeneous sets of studies may be systematically described and sources of confounding or bias can be elucidated.

## Conclusion

Overall, we believe it will be essential to use meta-analysis to address outstanding questions regarding HCV prevention. Because individual studies have failed to find evidence of specific protective factors for HCV in drug users (especially IDUs) and the etiology of HCV transmission in NIDUs remains somewhat vague, meta-analysis may prove to be highly useful in addressing endemic HCV in drug users. For instance, combining data across seroconversion studies would substantially increase the ability to detect consistent and statistically significant risk factors. Additionally, identifying in more detail the relationship between HCV prevalence and incidence and number of years injecting is essential for honing and targeting prevention efforts [34].

For the HCV Synthesis Project, the data set is complete and data analysis is in process. A report on HCV in NIDUs is published [13]; a preliminary analysis of the relationship of gender, duration and age to HCV in IDUs is in press [34]; and a report on the relationship of racial and ethnic status to HCV in IDUs is in preparation. Future analyses in preparation will focus on complex questions of variability in HCV prevalence and incidence in injection drug users. It is anticipated that this study will help to summarize the state of HCV knowledge, identify new research questions or those that need additional confirmation, and may point towards promising HCV prevention strategies.

## Abbreviations

The following abbreviations are used in the body of the text: HCV: Hepatitis C Virus; IDU: Injection Drug User; NIDU: Non Injection Drug User; MOOSE: Meta-analysis of Observational Studies in Epidemiology study group; PY: Person Years; HIV: Human Immunodeficiency Virus; HBV: Hepatitis B Virus; HAV: Hepatitis A Virus.

## Competing interests

The authors declare that they have no competing interests.

## Authors' contributions

HH designed the meta-analysis described here, with consultation from DDJ, SS, and PF. HH wrote the first draft of this manuscript. RKS subsequently revised and completed the manuscript. CLW, RS, and DDJ contributed to and revised this manuscript. SS and PF made revisions to the manuscript. EP conducted the statistical analyses for the quality scoring process. All authors read and approved the final manuscript.

## Pre-publication history

The pre-publication history for this paper can be accessed here:


